# Unveiling the oral-gut connection: chronic apical periodontitis accelerates atherosclerosis via gut microbiota dysbiosis and altered metabolites in apoE^−/−^ Mice on a high-fat diet

**DOI:** 10.1038/s41368-024-00301-3

**Published:** 2024-05-13

**Authors:** Guowu Gan, Shihan Lin, Yufang Luo, Yu Zeng, Beibei Lu, Ren Zhang, Shuai Chen, Huaxiang Lei, Zhiyu Cai, Xiaojing Huang

**Affiliations:** 1 Fujian Key Laboratory of Oral Diseases & Fujian Provincial Engineering Research Center of Oral Biomaterial & Stomatology Key Laboratory of Fujian College and University, School and Hospital of Stomatology, Fujian Medical University, Fuzhou, China; 2 Institute of Stomatology & Research Center of Dental and Craniofacial Implants, School and Hospital of Stomatology, Fujian Medical University, Fuzhou, China; 3 Department of Stomatology, Fujian Medical University Union Hospital, Fuzhou, China

**Keywords:** Cardiovascular diseases, Dental diseases

## Abstract

The aim of this study was to explore the impact of chronic apical periodontitis (CAP) on atherosclerosis in apoE^−/−^ mice fed high-fat diet (HFD). This investigation focused on the gut microbiota, metabolites, and intestinal barrier function to uncover potential links between oral health and cardiovascular disease (CVD). In this study, CAP was shown to exacerbate atherosclerosis in HFD-fed apoE^−/−^ mice, as evidenced by the increase in plaque size and volume in the aortic walls observed via Oil Red O staining. 16S rRNA sequencing revealed significant alterations in the gut microbiota, with harmful bacterial species thriving while beneficial species declining. Metabolomic profiling indicated disruptions in lipid metabolism and primary bile acid synthesis, leading to elevated levels of taurochenodeoxycholic acid (TCDCA), taurocholic acid (TCA), and tauroursodeoxycholic acid (TDCA). These metabolic shifts may contribute to atherosclerosis development. Furthermore, impaired intestinal barrier function, characterized by reduced mucin expression and disrupted tight junction proteins, was observed. The increased intestinal permeability observed was positively correlated with the severity of atherosclerotic lesions, highlighting the importance of the intestinal barrier in cardiovascular health. In conclusion, this research underscores the intricate interplay among oral health, gut microbiota composition, metabolite profiles, and CVD incidence. These findings emphasize the importance of maintaining good oral hygiene as a potential preventive measure against cardiovascular issues, as well as the need for further investigations into the intricate mechanisms linking oral health, gut microbiota, and metabolic pathways in CVD development.

## Introduction

Chronic apical periodontitis (CAP) is an inflammatory reaction caused by endodontic bacterial antigens.^1^ Host infection and immune responses are responsible for alveolar bone resorption in the apical region.^2^ Inflammatory products, cytokines, and oxidative stress factors are readily absorbed from the lesion site due to the lack of an epithelial barrier.^3^ Therefore, in addition to causing local inflammation, CAP may also lead to systemic inflammation.^4^ Endodontic infections are associated with cardiovascular disease (CVD), particularly initial endothelial damage.^5^

CVD is the leading cause of death worldwide [http://www.who.int], and the primary risk factor is atherosclerosis, which is caused by inflammation and lipid accumulation in the medium and large arteries.^6^ The detection of bacterial DNA in atherosclerotic plaques suggested that the development of atherosclerosis may be influenced by distant infection or direct infection of cells in the vessel wall.^7^ Several oral bacteria, including *Porphyromonas gingivalis* (*P. gingivalis*), have been detected in atherosclerotic plaques, and an increase in lesion size was observed after oral infection with *P. gingivalis* in apoE^−/−^ mice.^8^ Microbiological analysis of 47 teeth from patients with CAP revealed a high percentage of *P. gingivalis*, 40.4%.^9^ Furthermore, *P. gingivalis* infection of the dental pulp cavity of C57BL/6J mice resulted in arterial endothelial damage, and *P. gingivalis* was detected in the arterial smooth muscle tissue.^10^ This bacterium has the ability to induce apoptosis in smooth muscle cells through Toll-like receptor 2.^11^ In a previous study, CAP was induced by dental infection with *P. gingivalis* in apoE^−/−^ mice, which not only increased the percentage of atherosclerotic plaques but also altered the gut microbiota.^12^ Despite the lack of clarity regarding the underlying mechanisms involved, these findings suggest that both the gut microbiota and oral microbiota are associated with CVD and may contribute to the development of atherosclerosis.

Intestinal metabolites play a crucial role in gut microbiota-host interactions.^13^ Trimethylamine, bile acid, and short-chain fatty acids are associated with the gut microbiota and have essential roles in host metabolic and cardiovascular functions.^14–16^ Hence, metabolomics has become an increasingly promising tool for understanding the relationship between the host and gut microbiota.^17^ Liquid chromatography–mass spectrometry (LC–MS) is an extremely sensitive, specific, high-resolution metabolomics research technique with high throughput, resolution, and sensitivity. A metabolic profile can be used as a diagnostic, prognostic or therapeutic biomarker in clinical medicine, providing new insight into disease pathogenesis.^18^ Metabolomics provides functional information on microbiomes to complement sequence data on host and gut microbiota interactions.^19^ To understand disease pathogenesis, analyses of the gut microbiota and microbial metabolomics are increasingly being used in conjunction.^13^

In this study, we constructed a *P. gingivalis*-induced CAP model in apoE^−/−^ mice and carried out a multiomics analysis of feces via a combination of gut microbiota analysis via 16 S rRNA sequencing and microbial metabolomics analysis via liquid chromatography‒mass spectrometry (LC‒MS). Intestinal mechanical and mucosal barriers were also tested to evaluate the correlation between intestinal permeability and atherosclerosis. Multiomics analyses of the gut microbiota and microbial metabolism may help unravel the mechanisms by which CAP affects atherosclerosis.

## Results

### Increased atherosclerosis after endodontic infection by *P. gingivalis* in apoE^−/−^ mice

**Fig. 1:**
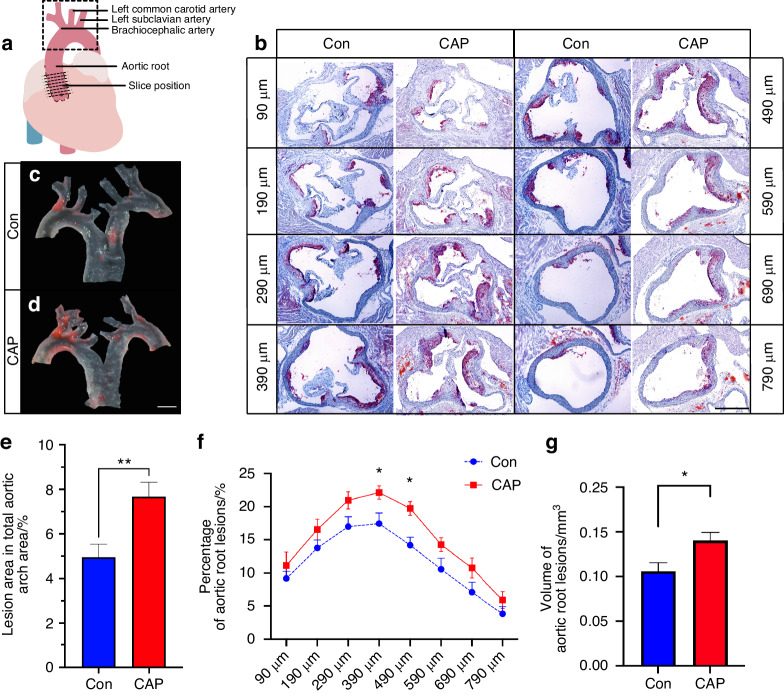
CAP exacerbates atherosclerosis in apoE^−/−^ mice. **a** Schematic of the dissection of the aortic root and aortic arch of the heart. The dotted straight line indicates the location of slicing at the aortic root, and the dashed rectangle indicates the location of the aortic arch sampling. **b** Representative images of frozen sections of the aortic root from frozen Oil Red O-stained sections from eight locations. Scale bar, 500 μm. **c** and **d** Representative images of Oil Red O-stained aortic arches. Scale bar, 1 mm. **e** Measurement of the percentage of atherosclerotic plaque at the aortic arch, *n* = 18–19; independent sample *t*-test; mean ± SEM; **P < 0.01. **f** and **g** Measurement of the percentage and volume of atherosclerotic plaques at the aortic root; *n* = 12; independent sample *t*-test; mean ± SEM, *P < 0.05

After 12 weeks of treatment, the range of periapical lesions increased in the CAP group (Supplementary Data 1a, b). To determine whether CAP exacerbates atherosclerosis in HFD-fed apoE^−/−^ mice, Oil Red O staining of aortic arch and aortic root sections was performed to evaluate the extent of atherosclerosis. The aortic arch and aortic root section sites were collected, as shown in Fig. 1a, and positive and negative controls were included to validate the specificity and reproducibility of the staining procedures. The Oil Red O-stained slices were collected from a total of 8 layers ranging from 90 to 790 μm from the aortic root for the CAP and control (Con) groups (Fig. 1b). Representative results of Oil Red O staining of the aortic arch (Fig. 1c, d) are shown, with a significant increase in the percentage of the lesion area in the aortic arch in the CAP group compared to that in the Con group (Fig. 1e; CAP, 7.674% ± 2.82%, Con, 4.949% ±2.53%, *n* = 18–19; *P* = 0.004). The percentage of the plaque area in the aortic root sections was greater in the CAP group than in the Con group (Fig. 1f; CAP, 22.16% ± 5.55%, Con, 17.45% ± 3.48% at 390 μm; *P* < 0.05, CAP, 19.74% ± 4.24%, Con, 14.17% ± 3.61% at 490 μm; *P* < 0.01, *n* = 12), and the plaque volume in the aortic root was greater in the CAP group than in the Con group (CAP, (0.134 ± 0.032) mm^3^, Con, (0.105 ± 0.033) mm^3^, *P* < 0.05, *n* = 12).

### Alterations in the gut microbiota of apoE^−/−^ mice after endodontic infection with *P. gingivalis*

**Fig. 2:**
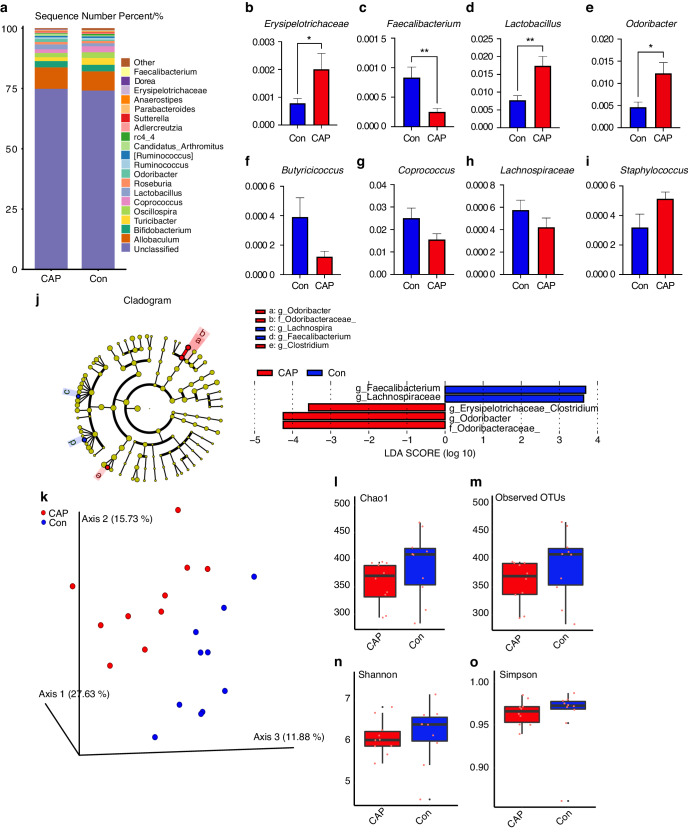
CAP leads to dysbiosis of the gut microbiota in apoE^−/−^ mice. **a** Comparison of the 20 richest abundances at the bacterial genus level between the two groups. **b**–**i** CAP leads to differentially expressed genera of gut microbes; *n* = 10, independent sample *t*-test; mean ± SEM, * indicates *P* < 0.05. **j** Enriched taxa in the CAP group (red) are indicated by negative log discriminant analysis (LDA) scores, and enriched taxa in the Con group have positive scores (blue). Only taxa with an LDA score (log 10) > ± 2 are shown. *n* = 10, LDA score > ±2 indicates *P* < 0.05. **k** Beta diversity of the gut microbiota from the two groups determined by Bray‒Curtis distance, ANOSIM test, *P* < 0.01, *n* = 10. Alpha diversity was demonstrated by the Chao1 (**l**), observed OTUs (**m**), Shannon (**n**), and Simpson (**o**) indices between the two groups

To assess the composition of the gut microbiota after endodontic infection by *P. gingivalis*, 16S rRNA sequencing was performed. This method enables the study of the microbial community structure and diversity by aligning sequences against the curated GREENGENES database. At the genus level, representative differentially abundant bacteria associated with atherosclerosis are shown (Fig. 2b–i). Linear discriminant analysis of effect sizes (LEfSe) was applied to distinguish the composition of the gut microbiota from the two groups, and 5 differentially abundant taxa (alpha = 0.05) were identified with a log discriminant analysis (LDA) score greater than 2.0 (Fig. 2j). At the genus level, *Lachnospiraceae* and *Odoribacter* were the most abundant in the CAP group, while *Faecalibacterium* and *Lachnospira* were the most abundant in the Con group (Fig. 2j). The results of principal coordinate analysis (PCoA) generated by the distance matrix calculated from Bray‒Curtis distances were compared for the bacterial communities, revealing the spatial separation of the samples from the CAP and Con groups (Fig. 2k). Axis 1 was the most significant contributor to the variation in the microbiota, accounting for 27.71%, while axis 2 explained 15.71%, and axis 3 explained 11.85% of the variation (Fig. 2k). The CAP and Con groups could be clearly distinguished by PCoA (*P* = 0.007 according to the Permanova test). The alpha diversity of the gut microbiota was evaluated using the Chao1 index, observed operational taxonomic units (OTUs), Shannon index, and Simpson index, all of which were lower in the infected group than in the Con group (Fig. 2l–o). To confirm the involvement of altered gut microbiota in the CAP-induced atherosclerotic process, a fecal microbiota transplantation (FMT) experiment was used to validate the contribution of CAP-induced alterations in the gut microbiota to atherosclerosis. The CAP-induced alteration of the gut microbiota was used as an independent factor and was investigated in apoE^−/−^ mice that were not treated with CAP. To prepare pseudosterile mice by quadruple antibiotic gavage,^20^ mice were divided into two groups by gavage of feces from the CAP and Con group mice for 12 weeks. Unsurprisingly, the gut microbiota of the CAP group of mice still exhibited an increased degree of atherosclerosis in pseudosterile mice (Supplementary Data 2a–c). In terms of beta diversity, the FMT-CAP group was closer in spatial proximity to the CAP group of samples, whereas the FMT-Con group was closer to the Con group, and the two types of samples were clearly distinguished from each other (Supplementary Data 2d). Similar to that of the CAP group, the alpha diversity of the FMT-CAP group was lower than that of the Con group (Supplementary Data 2e–g).

### Nontargeted metabolomics to detect altered intestinal metabolites in apoE^−/−^ mice after endodontic infection with *P. gingivalis*

**Fig. 3:**
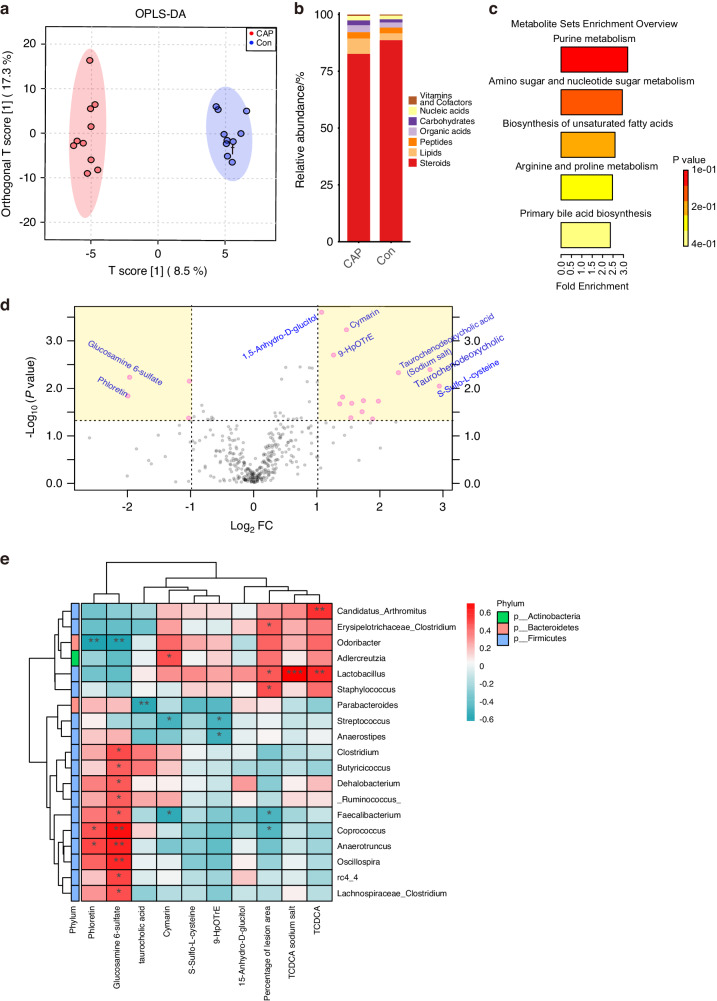
LC‒MS detection of altered metabolites in CAP-treated mice. **a** OPLS-DA score plot of the intestinal metabolites from the CAP and Con groups, *n* = 10, permutation test *P* ≤ 0.05, *Q*^2^ = 0.378. **b** Stacked column chart of the percentage of metabolites that play a biological role. **c** Overview of the metabolite set enrichment analysis based on the intestinal metabolites. **d** Volcano plot of the univariate analysis showing the differentially abundant metabolites. **e** Correlation clustering heatmap analysis of metabolites and the abundance of gut microbiota at the genus level. The columns show the different metabolites or the percentage of the lesion area, and each row indicates the different genera of the gut microbiota. The red and blue colors in the plot indicate high and low intensities, respectively; *n* = 10; *P < 0.05; **P < 0.01; ***P < 0.001

To investigate the effects of endodontic infection by *P. gingivalis* on microbial metabolites in apoE^−/−^ mice, we performed LC‒MS analysis of fecal samples collected at 19 weeks of age. The samples were first separated through liquid chromatography and subsequently introduced into a mass spectrometer for ionization and mass spectrometric analysis. The obtained data were subjected to multivariate and univariate statistical analysis to identify differentially abundant metabolites between the CAP and Con groups. Multivariate analysis using orthogonal partial least squares discriminant analysis (OPLS-DA) revealed a significant separation between the two groups (Fig. 3a). The quality of the model was validated using permutation tests (Supplementary Data 3, *P* ≤ 0.05, *Q*^2^ = 0.375). The histogram shows an increase in lipid metabolites and a decrease in steroids among the total metabolites in the CAP group (Fig. 3b). Metabolite set enrichment revealed a total of five metabolites enriched in metabolic pathways, including bile acid metabolism and biosynthesis of unsaturated fatty acids (Fig. 3c). Microbial metabolites are key players in host-microbiota crosstalk, and we further analyzed fecal samples from the CAP and Con groups of mice via univariate analysis. The results revealed that there were 18 metabolites with *P*-values < 0.05 and absolute fold changes greater than 2. In the CAP group, the levels of taurochenodeoxycholic acid (TCDCA) and the sodium salt TCDCA increased, while the levels of phloretin and glucosamine 6-sulfate decreased (Fig. 3d). To investigate the correlation among alterations in the gut microbiota, metabolites, and progression of atherosclerosis in the CAP group, correlation analyses were performed between the percentage of atherosclerotic lesions and fecal metabolites, as well as the relative abundance of gut microbes at the genus level. The results showed that nine alterations in metabolites were correlated with changes in 19 gut microbes at the genus level. Red and blue indicate positive and negative correlations, respectively (Fig. 3e, *n* = 10, * indicates *P* < 0.05; ** indicates *P* < 0.01; *** indicates *P* < 0.001). The percentage of the lesion area was positively correlated with *Erysipelotrichaceae*, *Lactobacillus*, and *Staphylococcus* but negatively correlated with *Faecalibacterium* and *Coprococcus*.

### Metabolomics of bile acids to detect altered intestinal metabolites in apoE^−/−^ mice after endodontic infection by *P. gingivalis*

**Fig. 4:**
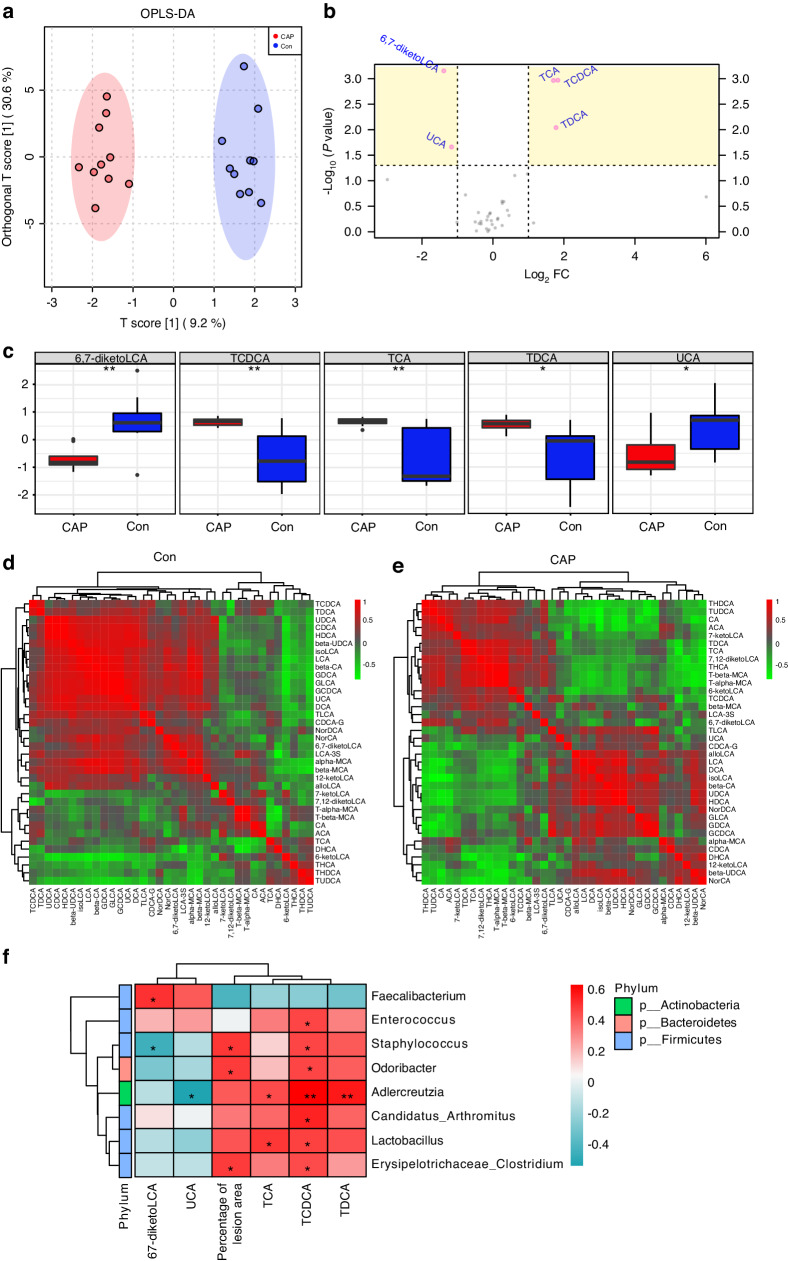
Altered bile acid metabolism in CAP-treated mice. **a** OPLS-DA score plot of the bile acid metabolites from the two groups; *n* = 10, *P* ≤ 0.01 in permutation test, *Q*^2^ = 0.767. **b** Volcano plot of univariate analysis showing the differentially abundant metabolites. **c** Box plot of differentially abundant metabolites, *n* = 10; *P < 0.05; **P < 0.01. **d** and **e** Clustered heatmap of correlations between 36 bile acids; the red and green colors in the plot indicate high and low intensities, respectively. **f** Correlation clustering heatmap analysis of bile acid levels and the abundance of the gut microbiota at the genus level. Each column refers to the different bile acids or the percentage of the lesion area, and each row indicates the different genera of the gut microbiota. The red and blue colors in the plot indicate high and low intensities, respectively; *n* = 10; *P < 0.05; **P < 0.01; ***P < 0.001

To further investigate the alterations in bile acid metabolism after CAP treatment, we performed a targeted metabolomics analysis of bile acid. When OPLS-DA was used for multivariate analysis, a pronounced distinction between the two sets became evident (Fig. 4a). To ensure the integrity of the model, permutation tests were conducted for validation purposes (Supplementary Data 4, *P* < 0.01, *Q*^2^ = 0.767). Metabolites exerting the most significant influence within the discriminant analysis procedure are those possessing variable importance in projection (VIP) value exceeding one. These specific metabolites substantially contributed to the discriminant analysis process and displayed marked dissimilarities across distinct subgroups. Metabolites labeled with their respective names within the yellow region exhibited a corrected *P*-value < 0.05 alongside a VIP value > 1. These particular metabolites exhibited notable dissimilarities among the subgroups and played a pivotal role according to OPLS-DA (Fig. 4b). Box plots of five bile acids with large differences in expression are shown to better illustrate the differences (Fig. 4c). In addition, the metabolic relationships of the 36 bile acids with each other also changed, and the investigation of gut bile acid metabolism yielded insightful results, as clearly represented in the Pearson correlation heatmap (Fig. 4d, e); the heatmap is colored according to the significance of the correlation between bile acids based on *P* values. The lightest green color indicates a *P*-value of 0, and the darkest red color indicates a *P*-value of 1. This heatmap serves as a comprehensive visual depiction of the interrelationships among diverse bile acid metabolites within the gut ecosystem. Each cell within the heatmap embodies a Pearson correlation coefficient, which effectively quantifies the degree and direction of associations between pairs of metabolites. Positive correlations, shown as warmer color tones, denote instances where an increase in one metabolite aligns with an increase in another, while negative correlations, represented by cooler tones, represent scenarios where an increase in one metabolite corresponds with a decrease in another. The color intensity serves as a quantitative indicator of correlation strength (Fig. 4d, e). We correlated differentially abundant bile acids and severity of atherosclerosis as environmental factors with differentially abundant gut microbes and showed that the percentages of atherosclerosis and TCDCA were positively correlated with *Staphylococcus*, *Lactobacillus*, and *Erysipelotrichaceae* (Fig. 4f).

### *P. gingivalis* impairment of the intestinal barrier after endodontic infection

Considering that alterations in the gut microbiota and metabolites promote the progression of atherosclerosis by altering intestinal barrier function, we examined intestinal barrier function.

**Fig. 5:**
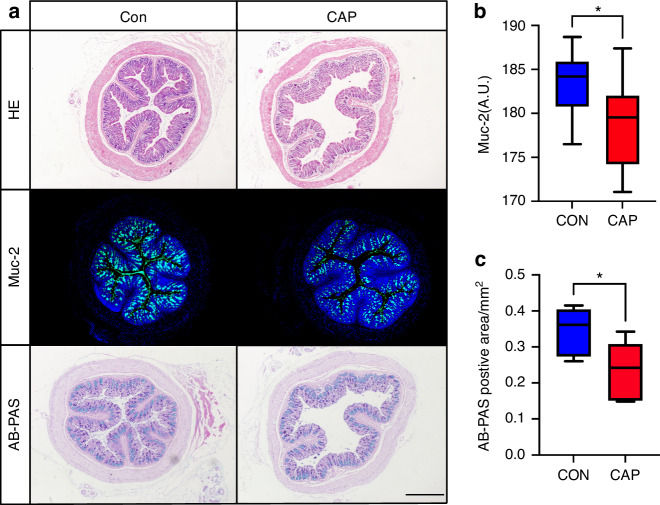
The intestinal mucus barrier was impaired in the apoE^−/−^ mice after CAP treatment. **a** Representative images of H&E staining, AB-PAS staining and Muc-2 immunofluorescence staining; scale bar, 500 μm; **b** Measurement of average Muc-2 fluorescence intensity, *n* = 9; independent sample *t*-test, mean ± SEM; *P < 0.05; **c** Measurement of the area of positive AB-PAS staining, *n* = 12; independent sample *t*-test, mean ± SEM, *P < 0.05

**Fig. 6:**
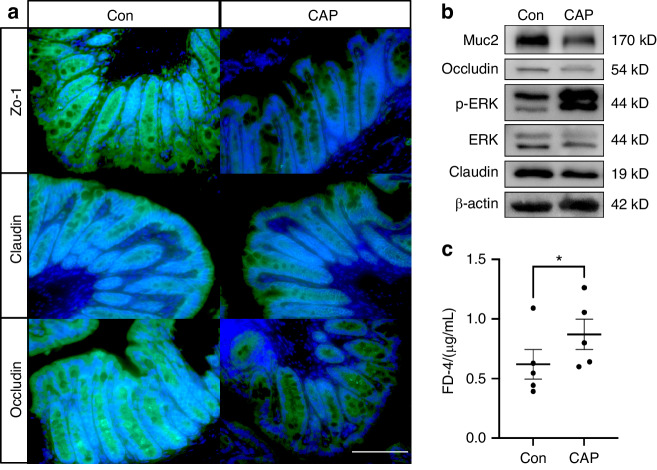
Increased intestinal permeability in apoE^−/−^ mice after CAP treatment. **a** Representative images of Zo-1, occluding, and claudin immunofluorescence staining. Scale bar, 100 μm; **b** Differences in protein expression in mouse colon tissue. **c** Intestinal permeability assay for FD-4 in serum; *n* = 5; paired samples *t*-test, *P* < 0.05

Colon pathological changes were evaluated by HE staining (Fig. 5a), and impaired epithelial integrity, decreased villus height and a decreased number of cup cells were observed in the CAP group to better determine alterations in the intestinal mucus. We performed immunofluorescence staining of intestinal mucin as well as Alcian blue-periodic acid Schiff (AB-PAS) staining (Fig. 5a). The expression of the intestinal Muc-2 protein was reduced in the CAP group; mean fluorescence intensity analysis was performed, which showed that the mean fluorescence intensity was reduced in the CAP group (Fig. 5b, *P* < 0.05). AB-PAS staining revealed a decrease in the number of intestinal cup cells, and quantitative analysis of the mucus revealed a decrease in the area of positive AB-PAS staining in the CAP group (Fig. 5c, *P* < 0.05). Intestinal epithelial paracrine junctional proteins were also examined by immunofluorescence, and the results showed that the fluorescence intensity of Zo-1, claudin, and occludin was significantly decreased in the CAP group (Fig. 6a). The results of Western blotting of intestinal tissues to analyze barrier function were consistent with the immunofluorescence results, which revealed a reduction in the intensity of Muc-2, Occludin and Claudin expression in the CAP group (Fig. 6b). Subsequently, we carried out in vivo experiments to detect changes in the intestinal permeability of mice in the CAP group by administering fluorescein isothiocyanate-dextran 4 (FD-4), which is a fluorescently labeled dextran with a molecular weight of 4 kD. Due to its relatively small molecular weight, FD-4 can be used as a model substance for solute transport across cell membranes, and the results showed that the serum level of FD-4 in the CAP group was greater than that in the Con group (Fig. 6c), indicating that the intestinal permeability of mice in the CAP group was increased. Moreover, we carried out a correlation analysis between intestinal permeability and atherosclerotic plaques in the aortic arches of the mice, and the results showed a positive correlation (Supplementary Data 5).

## Discussions

Infection can affect the development of atherosclerosis through two mechanisms: first, direct infection of cells in the vessel wall can increase the number of sites susceptible to lesions, and second, indirect infection at sites away from atherosclerotic plaques can activate the immune system, which in turn increases the systemic inflammatory state.^21^ Poor oral hygiene has been recognized as an established risk marker for CVD.^22^ Studies have demonstrated that increased expression of CAP-mediated cytokines, including IL-2 and IL-6, leads to an initial inflammatory response in the rat aorta.^23,24^ Furthermore, studies have shown irreversible changes in the liver, indicating that CAP has systemic effects on multiple organs.^24^ Previous experiments have revealed that CAP promotes atherosclerosis in apoE^−/−^ mice while altering the composition and diversity of the gut microbiota.^12^ Notably, FMT with an altered gut microbiota as an independent variable also exacerbated the degree of atherosclerosis in apoE^−/−^ mice, suggesting a causal link between the gut microbiota and atherosclerosis.^25^ However, the specific mechanisms through which the gut microbiota contributes to the development of atherosclerosis require further exploration. Based on the findings of the present study, it is possible that CAP impacts atherosclerosis by modulating gut microbiota metabolites and intestinal permeability.

The results indicated a statistically significant increase in the aortic arch in the CAP group (Fig. 1e, *P* < 0.01), suggesting that CAP causes an increase in the area of arterial endothelial injury in the apoE^−/−^ mice. The results showed that the atherosclerotic plaque volume in the aortic sinus was greater in the CAP group than in the Con group (Fig. 1f, g), and CAP may have increased the extent of lipid accumulation in plaques. These findings are consistent with the results of the CAP model in apoE^−/−^ mice fed a chow diet.^12^ However, there were some significant differences between these models and previous CAP models. First, the diet was different; this study used a high-fat diet instead of the previous chow diet. Second, to simulate the clinical situation and based on several infections in experimental periodontitis,^26^ constructed a CAP model by several rounds of infection. These results suggest that regardless of whether the apoE^−/−^ mice are fed a chow diet or an HFD, CAP can exacerbate atherosclerosis.

A HFD has been shown to affect the diversity and composition of the gut microbiota in humans and rodents,^27^ and considering that an HFD can alter the composition of the gut microbiota, this study aimed to investigate whether CAP affects the composition and diversity of the gut microbiota in apoE^−/−^ mice fed an HFD. To assess the effects of endodontic infection with *P. gingivalis* on the gut microbiota composition and diversity in association with HFD feeding, we performed 16S rRNA sequencing and analyzed the data using various bioinformatics tools. The top 20 altered genera are shown in Fig. 2a, and for ease of visualization, we selected eight of them for presentation via box plots (Fig. 2b–i). LEfSe was applied to the microbiota data of the CAP group and Con group, and four differentially abundant taxa were identified at the genus level (Fig. 2j). *Erysipelotrichaceae* and *Odoribacter* were found to be enriched, while *Faecalibacterium* and *Lachnospiraceae* were replete in the CAP group compared to the Con group (Fig. 2j). This result is consistent with clinical studies in which *Erysipelotrichaceae* was significantly enriched in the gut microbiota of patients with atherosclerotic CVD, heart failure syndrome with a reduced ejection fraction, and heart failure syndrome with a preserved ejection fraction.^28^ On the other hand, a decrease in *Faecalibacterium* abundance and an increase in *Lactobacillus* abundance were found in the gut microbiota of patients with clinical manifestations of atherosclerotic plaques with ischemic stroke,^29^ and the abundance of *Faecalibacterium* was negatively correlated with different markers of inflammation, such as high-sensitivity C-reactive protein and IL-6.^30^ The results showed that CAP increased the abundances of bacterial genera positively associated with atherosclerosis while decreasing those negatively associated with atherosclerosis. In terms of beta diversity, the gut microbiota of the CAP and Con groups were clearly distinguishable (Fig. 2k, *P* = 0.007 according to the PERMANOVA test). These findings suggest that CAP can still have significant effects on the composition of the gut microbiota even under HFD feeding. In terms of alpha diversity, the Chao1 and observed OTU indices represent the number of species in the microbiota, and the Shannon and Simpson indices represent the diversity of the microbiota (Fig. 2l–o). There was a reduction in the diversity and abundance of the gut microbiota after CAP treatment and HFD feeding, but the differences were not statistically significant (Fig. 2l–o, *P* > 0.05). Alpha diversity was significantly reduced in apoE^−/−^ mice fed an HFD,^31^ and considering previous findings,^12^ there was a relative increase in the diversity and abundance of the gut microbiota after CAP treatment in mice fed a chow diet. The most likely cause of the alteration in alpha diversity was the combination of CAP and HFD, and the effect of HFD on alpha diversity was greater than that of CAP. The most direct and commonly used research method to prove causality is FMT.^32^ After FMT treatment, the gut microbiota richness (Supplementary Data 2e–g) and diversity (Supplementary Data 2d) of the FMT-CAP group were more similar to those of the CAP group, suggesting that the CAP gut microbiota successfully colonized the gut of the FMT-CAP mice and that the percentage of atherosclerosis in the FMT-CAP group was greater than that in the FMT-Con group (Supplementary Data 2a–c). FMT treatment validates the involvement of CAP-induced alterations in the gut microbiota in promoting atherosclerosis. Notably, the resolution and accuracy of 16S rRNA sequencing may be limited, and additional techniques such as shotgun metagenomics or metatranscriptomics may provide a more comprehensive characterization of the gut microbiota. In summary, CAP may influence the process of atherosclerosis by increasing the abundance of harmful bacteria and decreasing the abundance of beneficial bacteria.

Metabolites produced by the gut microbiota play a crucial role in communication between the host and microbiota. According to the untargeted metabolomics analysis, all the samples included in the OPLS-DA were within the 95% confidence interval region of the ellipse, and the results revealed a strong separation effect between the two groups (Fig. 3a, *n* = 10, *P* ≤ 0.05). These results indicate that CAP is associated with an alteration in the composition of metabolites in apoE^−/−^ mice. The biological role of each metabolite was identified by annotating them with the KEGG database. The percentage of each biological role was then determined and presented as a stacked histogram, and the results showed an increase in metabolites related to lipid metabolism (Fig. 3b); it has been established that disorders of lipid metabolism play an essential role in the pathogenesis of atherosclerosis.^33^ The magnitude of metabolite alterations and that of some metabolites of interest were determined by calculating the fold change in metabolite abundance and *P*-value (Fig. 3d). In the CAP group, the levels of TCDCA and cymarin increased, while those of glucosamine 6-sulfate and phloretin decreased. The alteration in TCDCA observed in this study is similar to the effects of aging on the gut microbiota. High levels of serum TCDCA induced by aging activate TMAO-associated signaling pathways via hepatic FXR.^34^ Previous experiments also revealed increased serum levels of TMAO in mice in the CAP group,^25^ suggesting that CAP may influence the development of atherosclerosis by modulating the metabolism of bile acids in the gut microbiota and affecting the serum TMAO concentration. These findings are consistent with those of the metabolite set enrichment analysis, which revealed an alteration in primary bile acid synthesis in the CAP group (Fig. 3c). The CAP group presented high expression levels of primary conjugated bile acids, including TCDCA and taurocholate acid. Glucosamine 6-sulfate has the ability to reduce insulin resistance and proinflammatory effects by combining with amyloid-β.^35^ The concentration of phloretin, a dihydrochalcone flavonoid, was reduced in the CAP group (Fig. 3d); phloretin has been shown to have thrombogenic and atherosclerotic preventive effects by inhibiting thrombin-mediated leukocyte–platelet–endothelial interactions,^36^ and low doses of phloretin have also been found to optimize lipid metabolism and prevent the acceleration of atherosclerosis in streptozotocin-induced diabetic mice.^37^ The purpose of this study was to investigate the correlation between alterations in the gut microbiota and its metabolites induced by CAP and the development of atherosclerosis. The percentage of the lesion area and the nine candidate metabolites obtained by univariate analysis were used as environmental factors. These factors were subjected to correlation clustering heatmap analysis with CAP-induced alterations in the gut microbiota at the genus level (Fig. 3e). The red and blue colors in the graph represent the intensity of correlation from high to low. The percentage of the lesion area was found to cluster with that of the TCDCA and TCDCA sodium salt groups. Based on correlation and cluster analysis, the gut microbiota and its metabolites exhibited similar patterns, which was consistent with our hypothesis. The gut microbiota of the CAP group was enriched in *Erysipelotrichaceae*, *Lactobacillus* and *Staphylococcus* (Fig. 2d, e, i). These genera were found to be positively correlated with the severity of atherosclerosis or with deleterious metabolites. TCDCA was found to be positively correlated with *Lactobacillus* (Fig. 3e), and it was reported to be associated with increased systemic inflammation and intestinal permeability caused by a ketogenic diet.^38^ Additionally, another study showed increased levels of primary taurine-conjugated bile acids, including taurocholic acid and TCDCA, in mice treated with the *Lactobacillus casei Strain*.^39^ Notably, *Staphylococcus* was positively associated with the extent of atherosclerosis, and this result is consistent with research showing that *Staphylococcus* was enriched in four thrombus samples taken from patients undergoing thrombectomy.^40^ In contrast, a positive correlation was found between beneficial bacteria and beneficial metabolites. For instance, *Faecalibacterium* was positively correlated with glucosamine 6-sulfate, which is the functional group of enoxaparin responsible for the anti-inflammatory effect.^41^ There was also a negative correlation between beneficial metabolites and enriched genera in the CAP group. Of the more significant genera, *Odoribacter* was negatively correlated with both phloretin and glucosamine 6-sulfate (Fig. 3e). In summary, CAP leads to a decrease in beneficial gut microbes and metabolites and an increase in deleterious gut microbes and metabolites. The percentage of atherosclerotic lesions clustered with harmful metabolites and alterations in the gut microbiota were positively correlated with atherosclerosis.

Primary bile acid synthesis may play a crucial role in the promotion of atherosclerosis by CAP, as indicated by nontarget metabolomics data. Furthermore, the feces were subjected to bile acid metabolomics analysis. OPLS-DA clearly distinguished the composition of bile acids in the CAP group from that in the Con group (Fig. 4a), and the difference was statistically significant after permutation testing (Supplementary Data 4, *P* < 0.01, *Q*^2^ = 0.767). Subsequent screening for differentially abundant bile acids revealed increased levels of TCDCA, TCA, and taurodeoxycholic acid (TDCA) and decreased levels of UCA and 6,7-diketoLCA in the CAP group (Fig. 4b, c). The results of the correlation heatmap analysis revealed alterations in the correlations among the 36 bile acids, suggesting alterations in bile acid metabolism (Fig. 4d, e). Obeticholic acid was found to prevent the development of atherosclerosis by inhibiting intestinal cholesterol absorption, while TCA levels were found to be reduced by 71%.^42^ Carotid intima-media thickness (C-IMT), a proxy for subclinical atherosclerosis, was analyzed for correlation with patients’ serum metabolic profiles, and it was found that deoxycholic acid and TDCA levels were significantly greater in patients with abnormal C-IMT than in those with normal C-IMT.^43^ The levels of TCDCA and TCA were increased in the gingival tissues of patients with periodontitis and showed a positive correlation with periodontal clinical parameters.^44^ These results suggest that CAP-induced changes in bile acid levels may contribute to the extent of atherosclerosis by affecting intestinal permeability.

The barrier that separates the intestine from the external environment regulates interactions among intestinal contents, such as bacteria and the immune system. Mucin is crucial for intestinal barrier function, and Muc-2 is a key component of mucin in the colon. Muc-2 is produced by goblet cells, which form an internal and external mucus layer and cover the epithelial cell surface.^45^ Tight junction proteins, such as ZO-1, occludin, and claudin, are important components of the intestinal epithelial barrier and regulate the permeability of the intestinal epithelial barrier, preventing intestinal pathogenesis and increasing the permeability of tight junction proteins when damaged.^46^ Considering that bile acids may promote atherosclerosis by increasing intestinal permeability through disruption of the intestinal barrier, we stained tissue sections of the intestines and detected a decrease in villus height, intestinal mucus expression, intestinal mucin expression (Fig. 5a), and intestinal tight junction protein expression (Fig. 6a, b), suggesting that CAP causes damage to the intestinal mucus barrier and the intestinal mechanical barrier. Activation of the ERK1/2 signaling pathway is involved in mediating the downregulation of intestinal tight junction proteins,^47^ and LPS stimulation of intestinal epithelial cells also activated ERK1/2, leading to a decrease in tight junction proteins.^48^ This finding is consistent with our results showing that the ERK1/2 signaling pathway was activated in colonic tissue. An FD-4 in vivo assay was used to examine intestinal permeability in mice, and the results confirmed that CAP promotes intestinal permeability (Fig. 6c). Simultaneous dissection of the aortic arch was performed to quantify the percentage of the lesion area (Supplementary Data 5a, b), and a correlation analysis between the degree of atherosclerosis and permeability in mice revealed a strong positive correlation between the two parameters (Supplementary Data 5c, *P* < 0.05, *R* = 0.564). Consistent with our findings, impaired intestinal barrier function can result in bacterial translocation and the presence of bacterial products in circulation, potentially leading to atherosclerosis and chronic heart failure.^49^ Our results suggest that CAP may promote the development of atherosclerosis by increasing intestinal permeability.

**Fig. 7:**
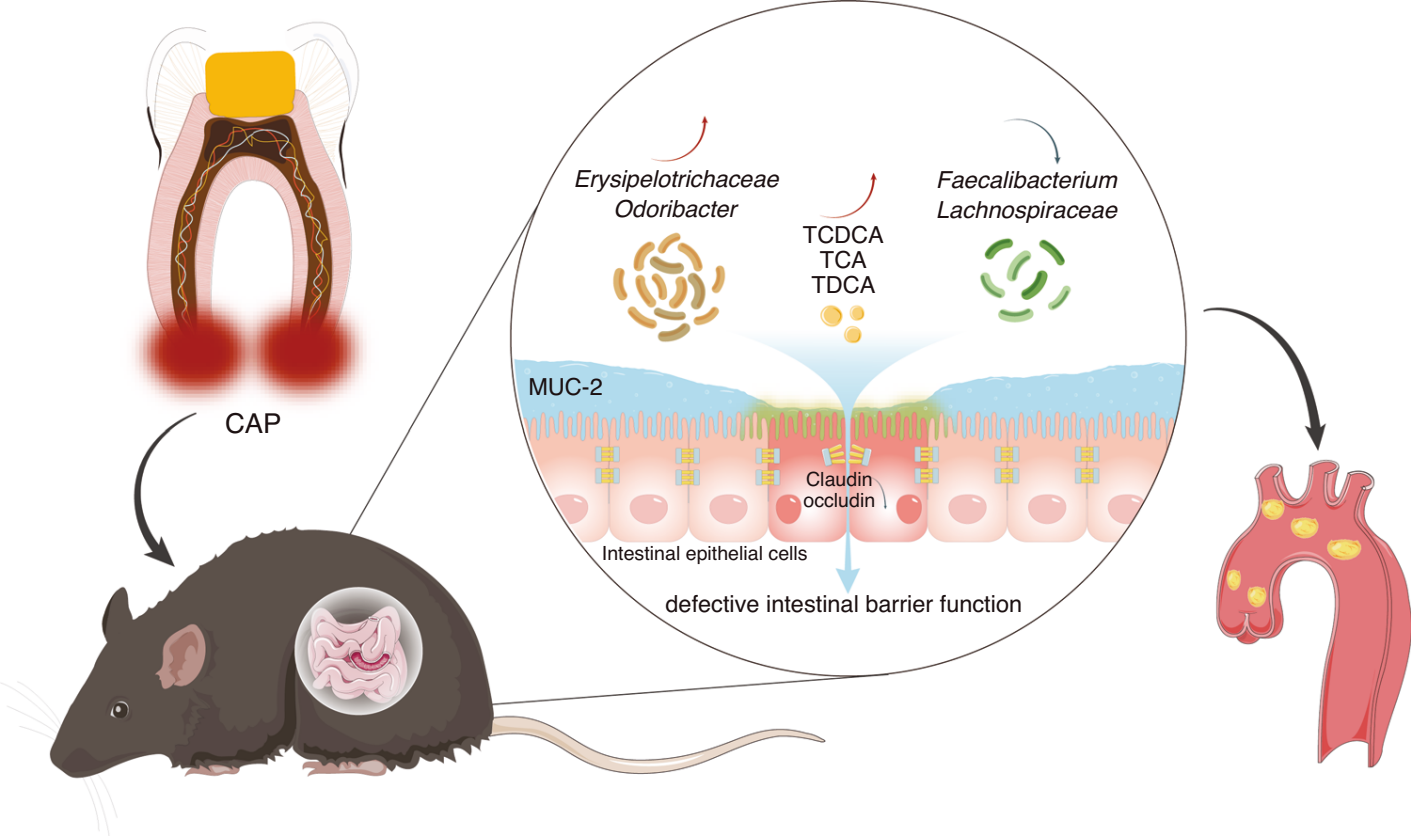
Schematic description of chronic apical periodontitis exacerbating atherosclerosis by affecting the gut microbiota and bile acid metabolism to promote intestinal permeability

In summary, this study revealed the complex interplay among oral health, the gut microbiota, metabolites, and CVD incidence (Fig. 7). These findings underscore the significance of maintaining oral hygiene and treating chronic apical periodontitis to promote cardiovascular well-being. Moreover, the findings of this study could lead to valuable insights into potential therapeutic strategies aimed at modulating the gut microbiota and metabolic pathways to mitigate the impact of CAP on atherosclerosis development. The limitations of this study include the fact that we did not detect the presence or absence of *P. gingivalis* infection in apical periodontitis or atherosclerotic plaques, which would have been interesting. Overall, this research contributes to our understanding of the multifaceted nature of CVD and offers prospects for novel interventions.

## Materials and methods

### Experimental design

The manuscript for this animal study was prepared following the Animal Research: Reporting of In Vivo Experiments (ARRIVE) guidelines. Seven-week-old male C57BL/6J apoE^−/−^mice (Cyagen Biotechnology Co., Ltd., Suzhou, Jiangsu, China) were raised in an experimental animal center in a specific pathogen-free environment with a 12-h light/dark cycle and free access to water and food and were fed an HFD after one week of adaptive feeding. All procedures were carried out according to the protocol approved by the Animal Care and Use Committee (protocol number: 2020-0041). The sample size was determined by G*power 3 (https://stats.idre.ucla.edu/other/gpower/, UCLA, USA), which referenced data from previous experiments.^12^ In this study, to achieve *P* < 0.05 and 95% power, *n* = 17 per group was needed. Considering the extended period, differences in diets, and the recommendations of the American Heart Association,^50^ the final decision on sample size was *n* = 19 in each group. Thirty-eight mice were randomly assigned to two groups (*n* = 19 per group) using random numbers generated by a computer. After one week of acclimatization, CAP was induced in the CAP group by *P. gingivalis* infection in the first and second molars of the bilateral maxillary region under anesthesia (0.1 mL per 10 g 1% pentobarbital; Sigma‒Aldrich, Shanghai, China). After the pulp chamber was exposed, a small piece of sterile cotton containing 0.2 µL of 10^8^ colony-forming units (CFU)/mL *P. gingivalis* (logarithmic growth phase, strain ATCC33277) was verified as ATCC33277 by 16S full-length sequencing against the NCBI database. Supplementary 6 a-b) was immediately inserted into the pulp cavity, followed by immediate temporary sealing with zinc oxide. The Con group was anesthetized only. All mice were fed an HFD to induce atherosclerosis. The specific operation schedule of the experiment is shown in the time flow diagram (Supplementary Data 1c).

#### Staining and quantification of atherosclerotic lesions in the aortic arch

The aortic arch was carefully dissected under a stereomicroscope (Leica Camera GmbH, Solms, Germany) and fixed in 4% paraformaldehyde for 24 h. The aortic arch was then opened along the lateral and medial curvatures. As described previously, the lesions were stained with Oil Red O (Sigma‒Aldrich, Shanghai, China).^51^ The images were obtained by stereomicroscopy and analyzed with Fiji (NIH, Bethesda, MD, USA; https://imagej.net/downloads). Considering individual differences in arterial plaque as well as the aortic arch, the percentage of atherosclerotic lesions was determined by dividing the area of red area plaques stained by Oil Red O by the area of the overall aortic arch after microdissection. One sample in the Con group could not be used to determine the extent of lesions in the aortic arch because of errors during microdissection.

#### Staining and quantification of atherosclerotic lesions in the aortic root

The evaluation of atherosclerotic lesions in the aortic root was performed mainly with reference to the recommended methodology.^51^ Cardiac tissues were removed from 4% paraformaldehyde solution, routinely dehydrated in a sucrose gradient (10% sucrose, 20% sucrose, 30% sucrose), embedded in OCT, and sectioned in a freezer to a thickness of 10 μm by a frozen slicer (NX70, Thermo Fisher Scientific, Sweden). The sections were retained starting at the point where three intact valves were observed at the root of the aorta^51^ (eight sections 90–790 μm from the aortic sinus were collected) and stored at −20 °C for Oil Red O staining. The frozen sections were rewarmed at room temperature and washed in distilled water to remove the embedding agent. The sections were washed with 60% isopropyl alcohol, stained with Oil Red O working solution, stained with 60% isopropyl alcohol under a microscope, and washed with water immediately. Glycerol gelatin was used to seal the sections. Oil Red O staining of the aortic root was used to evaluate the extent of atherosclerosis. Images were taken under an inverted microscope, and the results were measured and analyzed using ImageJ. Eight levels of atherosclerotic lesion area were outlined by ImageJ, and the atherosclerotic volume of each sample was calculated by calculating the area under the curve of the 8-level atherosclerotic area line graph band edges.

#### Micro-CT analysis

The paraformaldehyde-fixed jawbone samples were placed on a micro-CT system (μCT100, Seanco Medical, Bassersdorf, Switzerland) and scanned along the long axis of the specimen to obtain a continuous micro-CT image with a resolution of 1 024 × 1 024, a pixel size of 15 μm × 15 μm, and a layer spacing of 15 μm. The periapical bone resorption area was reconstructed using Mimics software.

#### 16S rRNA sequencing of the gut microbiota

Fresh fecal samples were collected at 19 weeks of age, and the samples were subsequently transferred to dry ice and stored at −80 °C. Microbial DNA was extracted using an EZNA® kit (Omega Bio-Tek, Norcross, GA, USA). A PCR thermocycler system (GeneAmp 9700, ABI, USA) was used to amplify the V3–V4 variable region (primers 338F and 806R), after which the products were extracted from 2% agarose gels using an AxyPrep DNA Gel Extraction Kit (Axygen Biosciences, Union, CA, USA). Further purification and quantification were conducted using QuantiFluor™-ST (Promega, USA) according to the manufacturer’s protocol. The GREENGENES database was used for microbiota annotation. LEfSe was used to identify bacteria that differed in abundance between the samples.^52^ The core diversity plug-in of QIIME2 was used to calculate diversity metrics and the alpha diversity index. The Bray‒Curtis distance was used as an index to explore beta diversity, which was determined via PCoA.^53^

#### Fecal microbiota transplantation

Two FMT groups were established: the CAP recipient group (FMT group receiving CAP, FMT-CAP group, *n* = 13) and the control recipient group (FMT group receiving Con, FMT-Con group, *n* = 13). The recipient mice in both groups were pretreated with a mixture of four antibiotics (ampicillin 1 g/L, neomycin 0.5 g/L, vancomycin 0.5 g/L, and metronidazole 1 g/L) for 3 days,^20^ and the feces of the mice in the donor group were collected to prepare the bacterial suspension, which was administered to the recipient group by gavage. In the first week, the frequency of gavage was maintained at two times a week, and then the dosage was changed to one once a week for 12 consecutive weeks. Fresh feces (200 mg) were collected from the donor mice, and 4 mL of sterilized saline was added. After mixing, the supernatant was left to stand, and the recipient mice were administered the supernatant by gavage.

### Metabolomics analysis

Mouse feces (100 mg) were ground separately in liquid nitrogen and then resuspended by vortexing with precooled 80% methanol. The samples were incubated on ice for 5 min and then centrifuged at 15 000×*g* for 20 min at 4 °C. Some of the supernatants were diluted to a final concentration of 53% methanol with LC‒MS-grade water. The sample was then transferred to a fresh Eppendorf tube and centrifuged at 15 000×*g* for 20 min at 4 °C. Finally, the supernatant was injected into the LC‒MS system for analysis.^54^ LC‒MS analysis was performed using a Vanquish UHPLC system (Thermo Fisher, Germany) and an Orbitrap Q ExactiveTM HF mass spectrometer (Thermo Fisher, Germany). The samples were injected onto a Hypesil Gold column (100 × 2.1 mm^2^, 1.9 μm) using a 17-min linear gradient at a flow rate of 0.2 mL/min. The eluents for the positive polarity mode were eluent A (0.1% FA, water) and eluent B (methanol). The negative polar mode eluents used were eluent A (5 mM ammonium acetate, pH 9.0) and eluent B (methanol). The Q ExactiveTM HF mass spectrometer was operated in positive/negative polar mode with a spray voltage of 3.2 kV, a capillary temperature of 320 °C, an intrathecal gas flow rate of 40 arb and an auxiliary gas flow rate of 10 arb. The raw data files were processed using Compound Discoverer 3.1 (CD3.1; Thermo Fisher, Germany) for peak matching, peak extraction, and quantification of each metabolite. The peak intensities were normalized to the total spectral intensity. The normalized data were used to predict molecular formulae based on additional ions, molecular ion peaks, and fragment ions. The peaks were subsequently matched against the mzCloud (https://www.mzcloud.org/), mzVault, and MassList databases to obtain accurate qualitative and relative quantitative results. The KEGG database (https://www.genome.jp/kegg/pathway.html), HMDB (https://hmdb.ca/metabolites), and LIPIDMaps database (http://www.lipidmaps.org/) were used for metabolite annotation. The R package MetaboAnalystR^55^ was used for data normalization and OPLS-DA. To bring the data closer to the normal parameters, MedianNorm, LogNorm, and AutoNorm were used. We applied univariate analysis of variance (*t*-test) to calculate the statistical significance (*P*-value).

#### Fluorescein isothiocyanate-dextran 4 (FD-4) assay for intestinal permeability

Intestinal permeability was measured by measuring FD-4 (60842-46-8, Sigma‒Aldrich, Shanghai, China) in 17-week-old mice. FD-4 was dissolved in saline solution and administered to mice by gavage at a concentration of 22 mg/mL in a 0.5 mL volume. After 5 h of gavage, the eyeballs were removed to collect blood, which was centrifuged at 10 000 r/min for 10 min at 4 °C. A total of 50 μL of the plasma was diluted in an equal amount of PBS (pH 7.4), and the concentration of FD-4 was determined by fluorescence spectroscopy using an excitation wavelength of 485 nm as a standard for serial dilutions and an emission wavelength of 528 nm. 7.4), and the concentration of FD-4 was determined using fluorescence spectroscopy at an excitation wavelength of 485 nm and an emission wavelength of 528 nm, with serially diluted samples used as standards. All samples and standards were measured three times.

#### AB-PAS staining

The experiments were performed according to the instructions of the AB-PAS kit (G1285, Beijing, Solarbio Technology Co.). Sections were stained in 3% acetic acid solution with 1% Alcian blue solution (pH 2.5) for 15–20 min. The sections were then rinsed in deionized water for 2 minutes and oxidized in 0.5% periodate solution for 5 min. The slides were rinsed and stained with Schiff’s reagent for 15 min, followed by hematoxylin staining.

#### Immunofluorescence staining to evaluate the mechanical and mucus barrier functions of the intestine

Paraffin-embedded tissue sections were deparaffinized and rehydrated, and citric acid was used to retrieve antigens at high pressure and temperature. After the goat sera were collected, they were incubated with primary antibodies (ZO-1, 1:50; 61-7300, Thermo Fisher Scientific, USA; Occludin, 1:50; 71-1500, Thermo Fisher Scientific, USA; Claudin, 1:50; Ab211737, Abcam, USA; and MUC-2, 1:50; Ab272692, Abcam, USA) overnight at 4 °C. A goat anti-rabbit fluorescent secondary antibody (1:1 000; Affinity Biosciences, S0008) was used. DAPI was used to visualize the b nuclei. Pictures were taken using an Olympus microscope (Olympus, Tokyo, Japan).

#### Western blot analysis

Twenty grams of mouse intestinal tissue was weighed, washed with cold PBS 2–3 times, and cut into small pieces. Then, 250 μL of RIPA lysis solution was added, and the sample was ground in a tissue-grinding apparatus. The homogenate was transferred to a centrifuge tube, shaken, and centrifuged at 12 000×*g* for 5 min, after which the supernatant was collected as the total protein solution. A BCA protein assay kit (Beyotime Biotechnology, Shanghai) was used to quantify the extracted proteins. After separation by SDS‒PAGE, the proteins were transferred to PVDF membranes. The PVDF membrane was blocked with 5% skim milk in Tris-buffered saline containing 0.05% Tween-20. The membranes were then incubated with primary antibodies against Muc-2, Occ, Cla, ERK1/2 (CST-4695, Cell Signaling Technology, USA), and p-ERK1/2 (CST-4370, Cell Signaling Technology, USA). The cells were incubated overnight at 4 °C and then incubated with the secondary antibody for 2 h at 37 °C. Protein bands were detected using enhanced chemiluminescence and imaged using the Clinx Chemiluminescence Imaging System. The intensity of the β-actin protein band was used as an internal reference.

#### Data analysis

GraphPad Prism 9 (GraphPad Prism Software, San Diego, CA, USA) was used to analyze the data and construct the graphs. The data were confirmed to be normally distributed, and the homogeneity of variance was determined before performing independent sample *t*-tests, including calculating the mean and standard deviation of the data. The significance level was set at *P* < 0.05.

### Supplementary information

Supplementary data

## Data Availability

The data associated with this article will be shared upon reasonable request to the corresponding author.
