# Supplementary material for: Unveiling the oral-gut connection: chronic apical periodontitis accelerates atherosclerosis via gut microbiota dysbiosis and altered metabolites in apoE−/− Mice on a high-fat diet
Source: Int J Oral Sci. 2024 May 13;16:39. doi: 10.1038/s41368-024-00301-3 (PMC11091127; doi:10.1038/s41368-024-00301-3)
Supplement: Supplementary file 1 — Supplementary data [file 41368_2024_301_MOESM1_ESM.pdf]

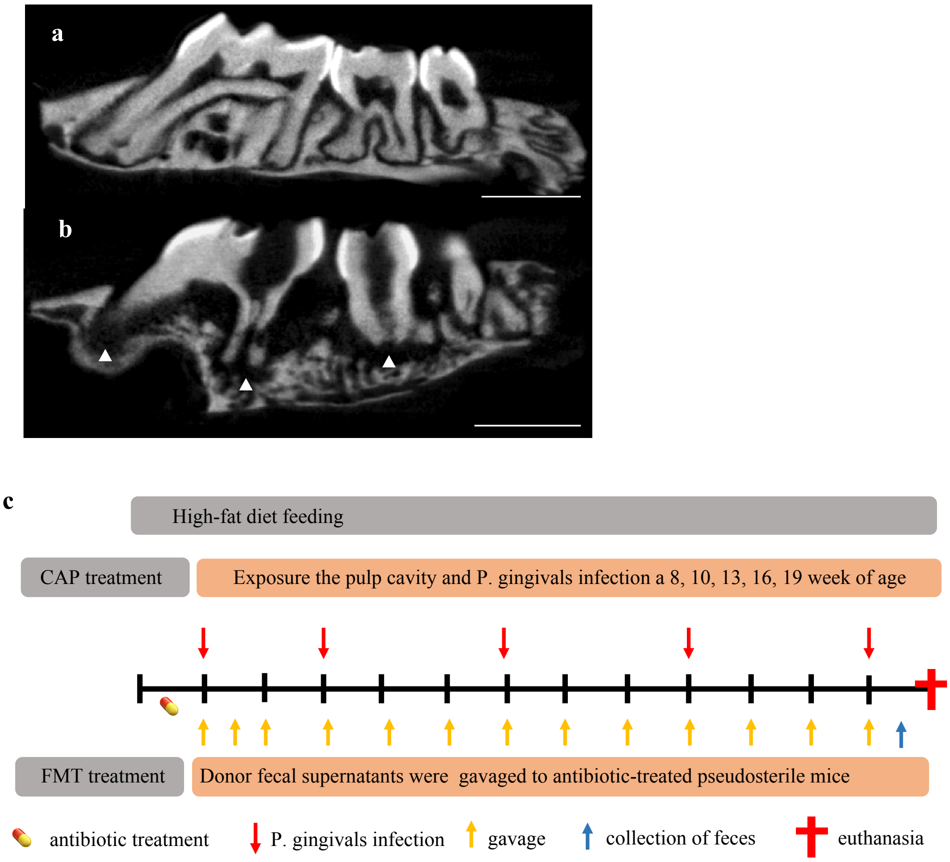

Supplementary data 1. Sagittal sections of the micro-CT images of the left maxillary first and second molars in the Con (a) and CAP (b) groups (scale bar: 1 mm). The white triangle indicates periapical lesions. c The time flow of the CAP treatments and the FMT experiments is schematically shown.

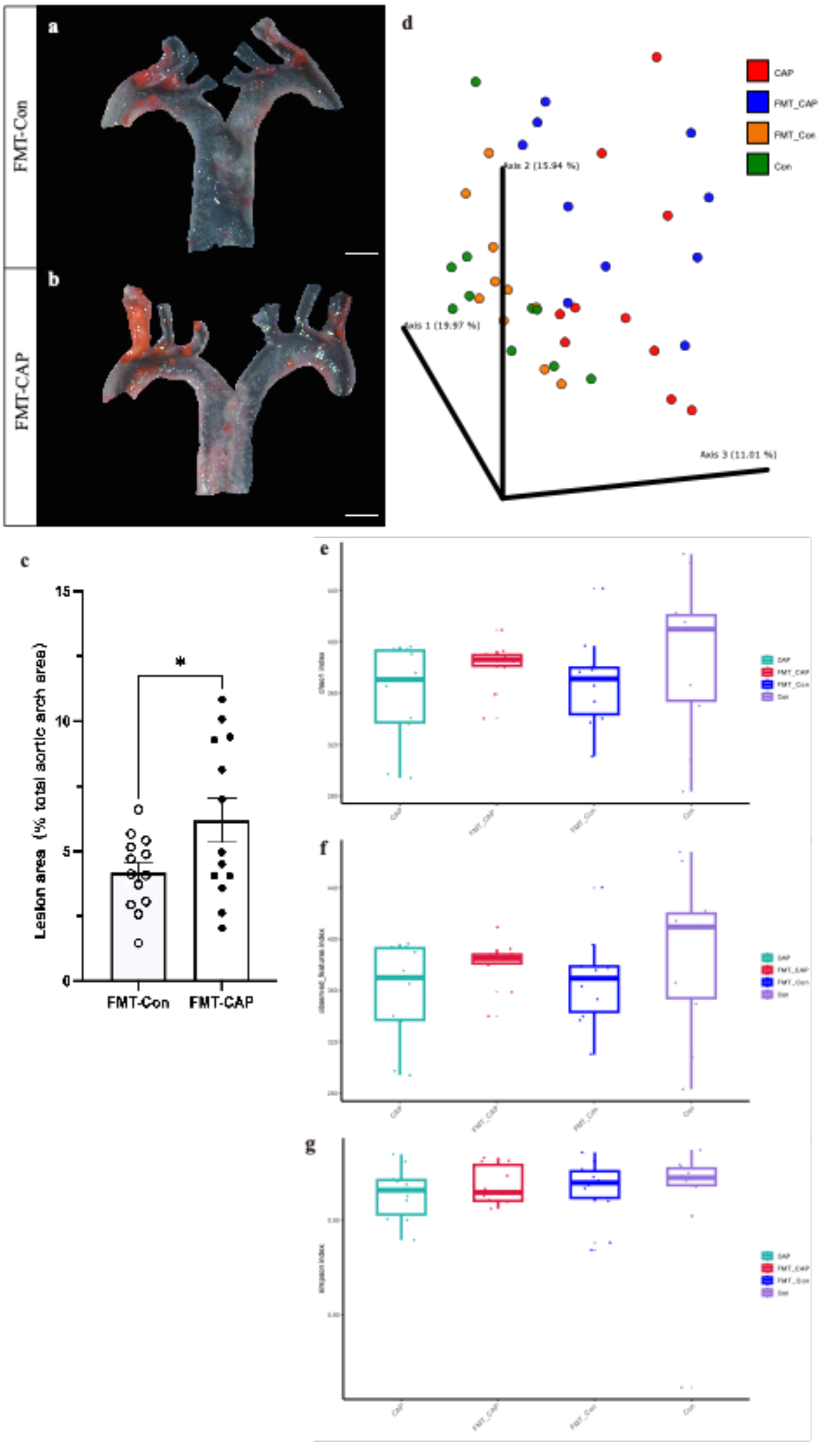

Supplementary data 2. CAP-induced changes in the gut microbiota are involved in promoting the development of atherosclerosis. a-b Representative images of the Oil Red O-stained aortic arches. Scale bar, 1 mm. c Measurement of the percentage of atherosclerotic plaque at the aortic arch; n = 13, independent samples t test; mean ± SEM, * indicates $P < .05$. d Beta diversity of the gut microbiota from the four groups determined by Bray-Curtis distance, n = 10. Alpha diversity was demonstrated by the Chao1 (e), Observed OTUs (f), and Simpson (g) indices among the four groups.

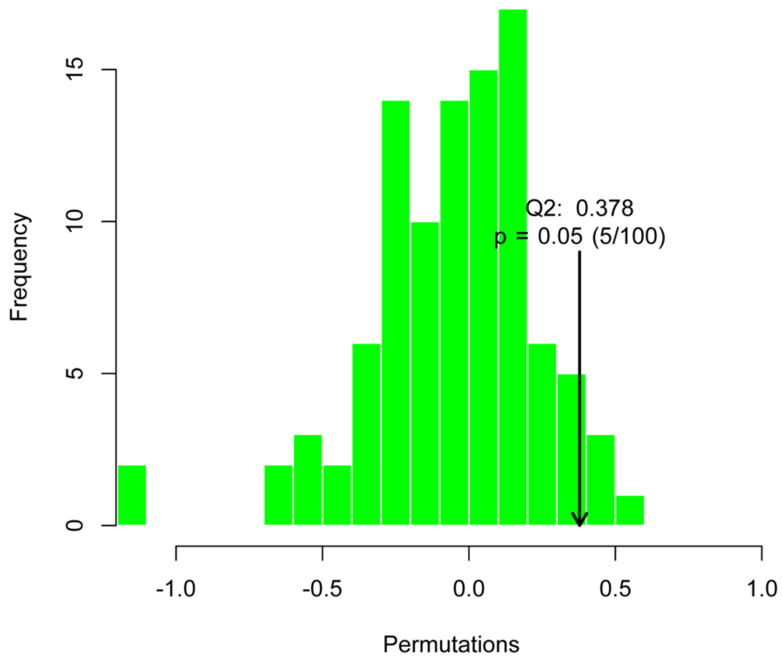

Supplementary data 3. Permutation of the results of the difference in OPLS-DA score plots was judged by nontargeted metabolomics (P ⩽.05, Q2 = 0.375 in the permutation test).

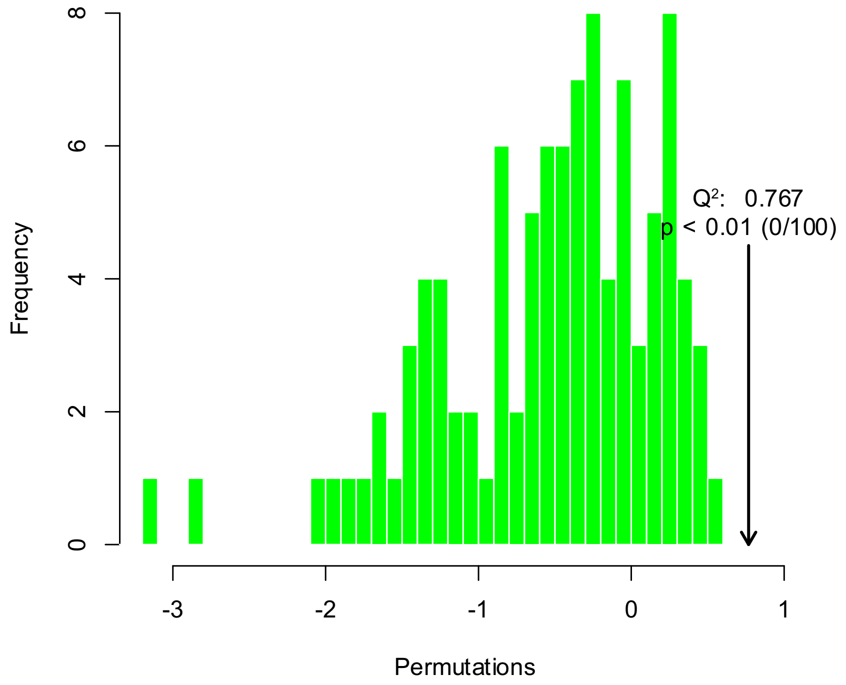

Supplementary data 4. Permutation of the results of the difference in OPLS-DA score plots was judged by bile acid metabolomics (P <.01, Q2 = 0.767 in the permutation test).

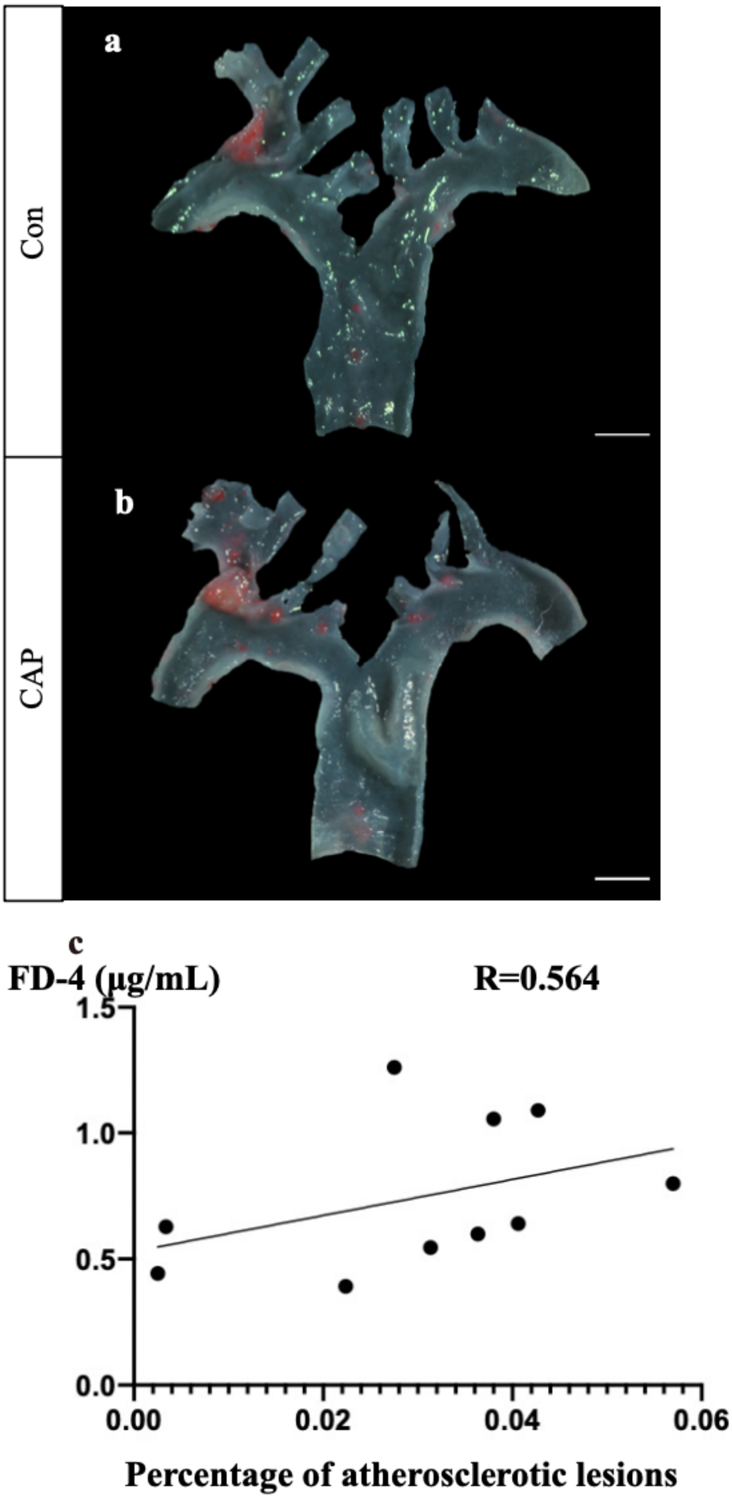

Supplementary data 5. Increased intestinal permeability positively correlates with the degree of atherosclerosis. a-b Representative images of the Oil Red O-stained aortic arches. Scale bar, 1 mm. c Correlation of FD-4 serum concentrations with the degree of atherosclerosis; Spearman correlation, n = 10, P <.05; correlation coefficient = .564.

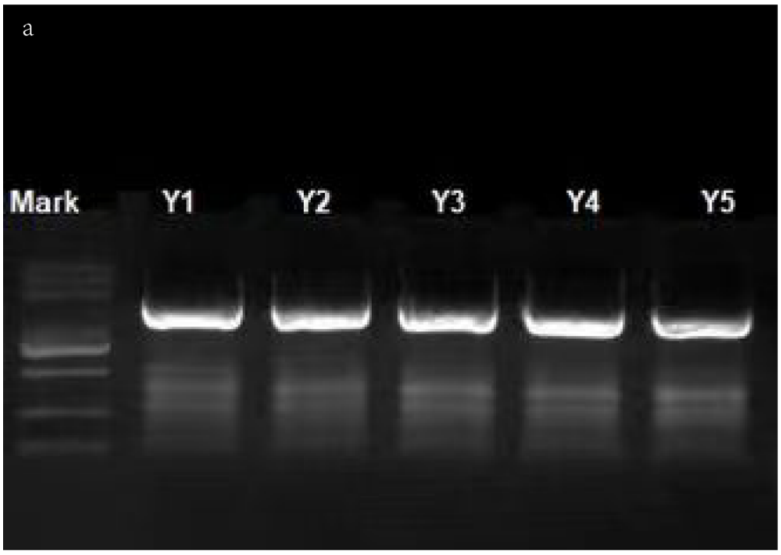

b

16S full-length sequence:

CCCTAGTGCGCCCCTTGCGGTTACGCCCTTCAGGTACCCCCGACTCCCATGGCTTG
ACGGGCGGTGTGTACAAGGCCCGGGAACGTATTCACCGCGCCATGGCTGATGCGC
GATTACTAGCGAATCCAGCTTCACGGAGTCGAGTTGCAGACTCCGATCCGAACTG
GGGAAGGGTTTAGAGATTCGCATCCGGTCGCCCGGTAGCTGCCCTTTGTCCCTCC
CATTGTAACACGTGTGTCGCCCCGGATGTAAGGGCCGTGCTGATTTGACGTCATC
CACACCTTCCTCGCGCCTTACGACGGCAGTCTCGGTAGAGTCCTCAGCGAAAACT
GTTAGCAACTACCGATGTGGGTTGCGCTCGTTATGGCACTTAAGCCGACACCTCAC
GGCACGAGCTGACGACAACCATGCAGCACCTACATAGACGCCCCGAAGGGAAGA
CGGTTTTCACCATCCGTCATCTACATTTCAATCCCGGGTAAGGTTCCTCGCGTATCA
TCGAATTAAACCACATGTTCCTCCGCTTGTGCGGGCCCCCGTCAATTCCTTTGAGT
TTCACCGTTGCCGGCGTACTCCCCAGGTGGATTACTTAACGCTTTCGCTGTGGAAG
CTTGACGGTATATCGCAAACTCCTAGTAATCATCGTTTACTGCGTGGACTACCAGGG
TATCTAATCCTGTTTGATACCCACGCCTTCGTGCTTCAGTGTCAGTCGCAGTATGGC
AAGCTGCCTTCGCAATCGGAGTTCCTCGTGATATCTATGCATTTCACCGCTACACCA
CGAATTCCGCCTGCCGCCACTGAACTCAAGCCCGGCAGTTTCAACGGCAGGCTGA
ACGTTGAGCGCTCAGGTTTCACCGCTGACTTACCGAACAACCTACGCACCCTTTAA
ACCCAATAAATCCGGATAACGCTCGCATCCTCCGTATTACCGCGGCTGCTGGCACGG
AGTTAGCCGATGCTTATTCTTACGGTACATTCAATGCAATACTCGTATCGCCCGTTATT
CCCGTATAAAAGAAGTTTACAATCCTTAGGACTGTCTTCCTTCACGCGACTTGGCTG
GTTCAGGCTCTCGCCCATTGACCAATATTCCTCACTGCTGCCTCCCGTAGGAGTCTG
GTCCGTGTCTCAGTACCAGTGTGGGGGATAAACCTCTCAGTTCCCCTACCCATCGTC
GCCTTGGTGAGCCGTTACCTCACCAACCAGCTAATGGGACGCATGCCTATCTTACAG
CTATAAATATTTCCTTGTAATATCATGCAATAATACAAGTGTATGCGGTTTTAGTCCGT
CTTTCAACGGGTTATCCCCCTCTGTAAGGCAAGTTGCATACGCGTTACGCACCCGTG
CGCCGGTCGCCATCAACCTTAGCAAGCTAAGATCATGCTGCCCCTCGACTTGCA

Supplementary data 6:

a Results of agarose gel electrophoresis assay after Pg recovery, b 16s full-length sequencing results.
